# Addressing gaps in the diagnosis of TB in children

**DOI:** 10.5588/ijtldopen.24.0239

**Published:** 2024-10-01

**Authors:** J. Hoffmann, C.R. Horsburgh

**Affiliations:** ^1^ Fondation Mérieux, Lyon, France;; ^2^ Boston University, Boston, MA, USA.

**Keywords:** tuberculosis, community, case finding, prevention, adolescent, epidemic

## Abstract

Since 2000, efforts to develop new treatments for TB have been promising, but diagnosing TB, especially in children, remains a challenge. This issue of the Journal includes the first in a series of articles related to TB in children, highlighting new diagnostic tests that do not rely on sputum, and have great potential for improving diagnosis and treatment initiation. Key to a reduction in TB prevalence, experts are engaging with communities to find undiagnosed cases and combat the stigma of TB. International collaborations are also central to integrating novel diagnostics and providing support for these vulnerable populations.

We live at a critical time for the human species. The COVID-19 epidemic taught us that mortal threats can arise and turn our everyday world upside down. It also sensitized us to the nature of airborne disease and the ease of transmission. This awareness has heightened the urgency for addressing the global TB epidemic, a scourge that has been a threat to human existence for millennia. TB was the reason The Union came into existence in 1920, but it was only recognized as a global emergency by WHO in 1993. Since then, over a million people have died of TB every year, dwarfing the mortality due to COVID-19.^1^ Fortunately, since 2000, there has been a concerted effort to develop new ways of treating TB. This is beginning to bear fruit, with over 20 therapeutic agents in the development pipeline or recently made available.^2^ It is reasonable to expect that we will soon have even more effective treatment regimens that are more acceptable to those who need them.^3^ However, the ongoing TB epidemic is not due to gaps in our ability to treat TB but rather due to gaps in our ability to diagnose it. Globally, over 3 million people with TB remain undiagnosed each year.^1^ This arises partially from our lack of accurate, rapid diagnostic tools, a problem that is particularly acute for children who struggle to produce sputum used for diagnostic tests. In 2022, there were an estimated 1.25 million children and young adolescents with TB, and 51% of those were not diagnosed; this gap is even more substantial in children under five years of age, where 58% were missed.^1^

A number of new diagnostics are currently being tested that could promptly and accurately identify TB in children and facilitate rapid treatment initiation. This issue of IJTLD OPEN introduces a new series on ‘TB diagnosis in children’, which addresses this gap.^4^ Experts in the field will present studies on promising diagnostic tests that do not rely on sputum, thus offering significant potential to enhance TB diagnosis in children. The first article, published in this issue, is an important contribution to the series and involves detecting TB in stools.^4^ Other articles in the series expand on the evaluation of TB blood tests as triage and diagnostic tools for pediatric TB and extra-pulmonary TB,^5^ and on exhaled breath condensate–lipoarabinomannan (EBC-LAM).^6^ The series will also include a review describing the global landscape of non-sputum-based diagnostic tools evaluated for childhood TB, and on gaps and future investment in finding and diagnosing TB in children, with a focus on rolling out new technologies. Too often, we see the development of new medical tools that are not promptly made available to the populations in greatest need. For both HIV and COVID-19, the rollout of new treatments and vaccines was delayed for people in low-resource settings. It is critical that we learn from these experiences and do not repeat the mistakes of the past.

Even more important than new diagnostics is community mobilization to engage populations in finding persons with undiagnosed TB. Community engagement is the only way to find the 30% of persons with TB who are never diagnosed and, as a result, continue to spread TB to contacts.^7^ Many of these people are not symptomatic and are surprised to learn that they pose a risk to their community. Others may be aware of their illness, but are deterred from seeking care because of the severe and ancient stigma of TB. Stigma has multiple effects that perpetuate the TB epidemic, including denial of illness, delayed presentation for care, suboptimal adherence to treatment and shunning by family and friends.^8,9^ Efforts to combat stigma are essential to bringing the TB epidemic under control, and community mobilization and education are proven ways to overcome this stigma.^10,11^ Fortunately, community engagement and mobilization programs have now come to the fore. The engagement of civil society and community leaders is essential to their success. Several studies have demonstrated that community mobilization, paired with broad screening programs, can make impressive reductions in the prevalence of TB.^12–14^ Community engagement in Uganda, Zimbabwe and Vietnam, followed by multi-year screening and treatment, have reduced TB prevalence by 43–68% in only a few years.^12–14^ Such projects, however, are resource-intensive and need to be tailored to local conditions. Th series on TB diagnosis in children will add important examples of community mobilization to our experience. Examples of successful engagement include the detection of TB infection and treatment uptake in children from the APRECIT Project in Cameroon and Madagascar.^15^ Examples like these demonstrate that culturally tailored programs can engage communities and achieve the goal of eliminating TB from their midst. Even more importantly, such engagement raises community awareness of TB and all aspects of health, allowing community members to achieve ‘healthy lives and well-being for all’ at all ages.^16^ At the 2023 World Conference on Lung Health, WHO released its updated Roadmap towards ending TB in children and adolescents.^17^ The articles in this series address several of the Roadmap’s key actions including ‘Implement and expand interventions for prevention’, ‘Scale up child and adolescent TB case finding and treatment’, ‘Implemented integrated family- and community-centered strategies’ and ‘Encourage research focuses on TB in children and adolescents’.

International scientific collaboration will play a key role in achieving the WHO's goals in the fight against infectious diseases. In 2008, the Mérieux Foundation (Lyon, France) set up the Global Approach to Biological Research, Infectious Diseases and Epidemics in Low-income Countries (GABRIEL), a network for sharing knowledge and field experience in the areas of antimicrobial resistance, acute respiratory infections and TB.^18^ The network currently comprises 16 countries and 22 members sharing their expertise on epidemiological surveillance and basic and applied research. In the field of TB, the foundation aims to help health authorities make strategic decisions on prevention, diagnosis and care. Projects are developed in partnership with and in support of National TB control programs, aiming to integrate diagnostic innovations into public health interventions, providing support to families affected by this disease. Since 2016, several projects have been launched within the GABRIEL network with the common aim of improving the diagnosis and management of TB disease, as well as screening for TB infection (the silent form of TB). The publications in this series will highlight this international scientific collaboration and demonstrate the value of local medical, scientific and socio-anthropological research. By bringing together key community and political players we aim to address TB and provide effective support for vulnerable populations.
